# Electrochemical Biosensors for Detection of Foodborne Pathogens

**DOI:** 10.3390/mi10040222

**Published:** 2019-03-28

**Authors:** Zhenguo Zhang, Jun Zhou, Xin Du

**Affiliations:** College of Life Sciences, Key Laboratory of Food Nutrition and Safety, Key Laboratory of Animal Resistance Biology of Shandong Province, Shandong Normal University, Jinan 250014, China; zhangzhenguo201@163.com (Z.Z.); junzhou@sdnu.edu.cn (J.Z.)

**Keywords:** electrochemical biosensor, pathogen, food, detection

## Abstract

Foodborne safety has become a global public health problem in both developed and developing countries. The rapid and precise monitoring and detection of foodborne pathogens has generated a strong interest by researchers in order to control and prevent human foodborne infections. Traditional methods for the detection of foodborne pathogens are often time-consuming, laborious, expensive, and unable to satisfy the demands of rapid food testing. Owing to the advantages of simplicity, real-time analysis, high sensitivity, miniaturization, rapid detection time, and low cost, electrochemical biosensing technology is more and more widely used in determination of foodborne pathogens. Here, we summarize recent developments in electrochemical biosensing technologies used to detect common foodborne pathogens. Additionally, we discuss research challenges and future prospects for this field of study.

## 1. Introduction

In the 21st century, foodborne diseases are particularly problematic. The development of science and technology and economic progress has been unable to effectively control the spread of foodborne diseases, which are instead showing an upward trend [1,2]. Food safety-related poisoning incidents occur frequently around the world and the incidence of foodborne diseases is high, regardless of country. The diseases caused by foodborne pathogens can be divided into four categories. The first is food poisoning, which refers to acute or sub-acute diseases that occur after eating food contaminated with toxic or hazardous substances [3,4]; the second is allergic diseases associated with food [5,6,7,8]; the third kind includes infectious diseases (dysentery) [9,10], zoonotic diseases (foot-and-mouth disease) [11,12] and so on; the last is disease characterized by chronic toxicity, caused by long-term ingestion of a large amount of certain toxic and harmful substances [13,14]. There is no doubt that foodborne diseases have become a global public health problem affecting everyone. It is difficult to evaluate the global incidence of foodborne disease, however, according to CDC 2011 estimates, one in six Americans get foodborne disease, 128,000 are hospitalized, and 3000 die of foodborne diseases annually [15,16]. A great proportion of these cases are due to the contamination of food and drinking water [17]. There are many kinds of pathogens that are capable of producing toxins causing foodborne diseases [18,19], among them *Escherichia coli*, *Vibrio cholerae*, *Bacillus cereus*, *Staphylococcus aureus* and *Clostridium perfringens* are common [20,21].

Routine detection process of pathogens includes non-selective and selective enrichment culture, plate separation, pure steps, biochemical reaction and serological identification, which are cumbersome, time-consuming and laborious [22,23,24,25]. The traditional technique is unable to meet the need of food safety supervision and rapid diagnosis of food pathogens [26,27,28]. In recent years, some rapid detection techniques were established with the development of biotechnology, such as detecting certain bacteria, bacterial automatic identification system and point-of-care technologies [29,30]. However, these methods also still have some limitations. Most of these techniques still require such steps as purifying cultures of bacteria and enriching bacteria. Furthermore, there may be more than one pathogen and microorganism in the food [31,32,33], hence, how to detect multi-target microorganisms at the same time by separating and enriching the pathogens in the food samples has increasingly become the focus of food microbial testing [34,35]. Therefore, the development of rapid detection of foodborne diseases has no time to delay.

At present, biosensing technology has more applications due to its advantages of unique sensitivity, low detection limit, and simple operation. Compared with traditional analytical methods, biological sensing technology has irreplaceable advantages: the first one is real-time, which can make mutual interactions with biological macromolecules and analysis using the changes that occur every moment of the process; the second is speediness, as the process takes only 5–15 min, and a large number of samples can be measured in a short time; the third is specificity and detection of other non-specific molecules in the sample; the last is simplicity, such that large molecules do not need to be labeled. The emerging electrochemical biosensing technique has been developed and applied to the microbial analysis of foodborne pathogens in a much shorter time, with high sensitivity and selectivity comparable to the conventional methods, which makes the idea of rapid detection of foodborne pathogens possible [36,37,38].

## 2. The Principle of Electrochemical Biosensors

**Figure 1:**
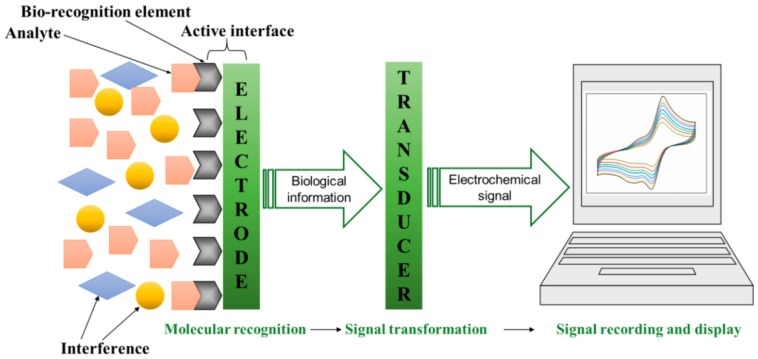
A schematic representation of the electrochemical biosensor. After the analyte contacts a recognition element on the surface of the biosensor, physical or chemical changes yield a reaction that is transformed into an electrochemical signal. This information can be further processed to determine the concentration of the pathogen and changes in the composition of the analyte.

The bio-recognition element is the core component of the electrochemical biosensor which was fixed on the surface of the electrode by physical or chemical method. The biosensor can selectively identify the target molecule and capture it onto the electrode surface, owing to the specific recognition function of bio-recognition element with the substance to be tested. As the main body of the signal converter, the electrode can derive the identification signal generated on the surface of the electrode and convert it into an electrical signal, including current, voltage, and resistance, which can be measured and analyzed in order to achieve qualitative or quantitative analysis of the analysis target. The operating principle of electrochemical biosensor is shown in Figure 1.

Electrochemical biosensors can be classified into amperometric, impedimetric, potentiometric and conductometric biosensors according to the observed data type, such as current, impedance, potential and conductance, respectively [37,38]. Electrochemical biosensors were the first reported type of commercialized biosensors in the history of biosensor development. The preparation of electrochemical-active interference is the crux for the superior reported biosensors developed to date [39]. However, electrochemical biosensors certainly possess disadvantages similar to other biosensors. Among the limits of electrochemical biosensor, the immobilization of bio-recognition element without denaturation or random orientation is the most insurmountable. Hence, most of the biosensors take advantage of self-assembled monolayer (SAM) modified gold electrode surfaces because they could supply favorable substrates and binding sites for bio-recognition element via the chemical groups (such as salines, thiols, acid, disulphides, or amines) in the surface of electrode [40]. According to the number of publications about electrochemical biosensors over the recent years, we can also declare that electrochemical biosensing technology is one of the most promising techniques within the field of foodborne pathogen detection.

## 3. Detection of Foodborne Pathogens Using Electrochemical Biosensing Techniques

In recent years, an increasing number of researchers focused on the detection of foodborne pathogens using electrochemical biosensing techniques. Therefore, in this review we summarize recent developments of electrochemical biosensors used to detect common foodborne pathogens. The detection methods, materials used and performance of electrochemical biosensors for foodborne pathogens are shown in Table 1.

### 3.1. Escherichia coli

*Escherichia coli* (*E. coli*) was discovered by Escherich in 1885, and had been considered a non-pathogenic bacterium and a normal part of gut flora for a long period of time [41]. Around the middle of the 20th century, it was recognized that some special serotypes of *E. coli* were pathogenic to humans and animals, especially to infants and young animals, and often cause severe diarrhea and sepsis [42]. Human are likely to be infected with *E. coli* by drinking contaminated water or eating unripe foods (especially beef, burgers and roast beef). In addition, a person whose hygiene is poor may be infected by human transmission, or by eating food contaminated with feces [43,44]. Therefore, detection of *E. coli* in our diet is vital for our health.

The reports of electrochemical biosensors for detection of *E. coli* are plentiful in foodborne pathogens [45]. As early as 2003, R. Mikkelsen et al. [46] have published screen-printed sensor arrays for the rapid determination of four *E. coli* subspecies (*E. coli* B, *E. coli* Neotype, *E. coli* JM105 and *E. coli* HB101). DNA biosensors are an effective means for detection of *E. coli*. For example, DNA nanopyramids were used by Leong et al. [47] to anchor *E. coli* lipopolysaccharides, lysate, and whole bacteria. Huang et al. constructed a simple, label-free, and low-cost electrochemical biosensor for highly sensitive detection of *E. coli,* based on rolling circle amplification (RCA) coupled with peroxidase-mimicking DNA enzyme amplification. The *E. coli* could specifically bind to the G-quadruplex units in an aptamer-primer probe, which leads to the formation of numerous G-quadruplex oligomers on electrode. Owing to the K^+^ and hemin on the electrode, the G-quadruplex/hemin complexes were able to generate extremely strong catalytic activity toward H_2_O_2,_ and then strong electrochemical response could be detected. Recently, Ranjbar et al. prepared polyanilinated amino-functionalized metal–organic frameworks (MOFs) to link amine-modified DNA aptamer by glutaraldehyde (GA). The fabricated biocomposite was used to capture *E. coli* O157:H7 and methylene blue (MB) as electrochemical indicators in differential pulse voltammetry detection.

Label-free electrochemical biosensors also were developed for detection of *E. coli*. Using graphene wrapped copper(II)-assisted cysteine hierarchical structure (rGO-CysCu), Malhotra et al. [48] fabricated an immune-electrode which realized that *E. coli* O157: H7 cells could be differentiated from the non-pathogenic *E. coli* and other bacterial cells. Another label-free electrochemical biosensor was developed by Wang et al. [49] based on a novel 3D Ag nanoflower. The [Fe(CN)_6_]^3−/4−^ was used as the redox probe to detect the resistance changes when *E. coli* O157:H7 was captured by the biosensor.

**Figure 2:**
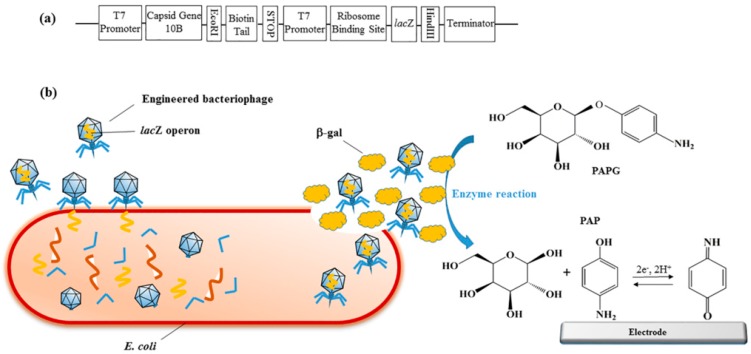
Scheme representation of electrochemical detection of *E. coli* using engineered phage. (**a**) The designed construct of genome of T7lacZ phage. (**b**) Specific capture and infection of *E. coli* by T7lacZ phage resulted in the release and overexpression of enzyme β-gal. PAPG was catalyzed by β-gal into an electroactive species PAP that can be quantified by electrochemical device. Reprinted with permission from [50]. Copyright (2017) American Chemical Society.

Furthermore, as shown in Figure 2, Nugen et al. [50] tactfully used T7 bacteriophages engineered with lacZ operon to infect *E. coli* and trigger the overexpression of beta-galactosidase (β-gal). The β-gal would catalyze the 4-aminophenyl-β-galactopyranoside (PAPG) as a substrate and release the electroactive species paminophenol, which could be detected by electrochemical method. Tan et al. [51] introduced amino groups by decorating the surfaces of CdS@ZIF-8 muti-core-shell particles through polyethyleneimine, in order to absorb the anti-*E. coli* O157:H7 antibody. The Cd(II) ions would release from CdS@ZIF-8 after the target was captured, and then the current could be detected by differential pulse voltammetry.

### 3.2. Vibrio cholerae

*Vibrio cholerae* is the pathogen of human cholera which is one of the ancient and widespread epidemic diseases. *Vibrio cholerae* has caused many pandemics in the world, mainly characterized by severe vomiting, diarrhea, water loss, and high mortality [52,53,54]. Therefore, it belongs to international quarantine classifications of infectious diseases.

The first *Vibrio cholerae* electrochemical biosensor was developed by Rao et al. [55] based on disposable screen-printed electrodes (SPE) to adsorb the polyclonal antibodies (PAb) of *Vibrio cholerae*. When bacterial cells bound to the surface of electrode, the antibodies conjugated to alkaline phosphatase (ALP), as the enzyme tracer catalyzed 1-naphtyl phosphate as its substrate, and then gave an electroactive product which could be detected via an amperometric method. This amperometric biosenor was further applied to study spiked water samples detecting as few as 8 CFU mL^−1^ in sea water and 80 CFU mL^−1^ in tap water through an enrichment step [56]. A similar amperometric biosensor for the detection of *Vibrio cholerae* was described by Doblin et al. using a biotinylated PAb, immobilized on neutravidine modified surface of SPE [57]. A one-step label-free biosensor for *V. cholerae* detection was developed using antibodies covalently immobilized on a CeO_2_ nanowire-modified microelectrode to capture the targets. The resulting biosensor was detected by impedance analysis with [Fe(CN)_6_]^3−/4−^ as the redox probe [58].

### 3.3. Bacillus cereus

*Bacillus cereus* (*B. cereus*), a species of the genus *Bacillus*, has close contact with humans and can cause food poisoning [59]. Many foods, especially leftovers that have been improperly refrigerated, can cause this type of diarrhea [60,61]. The symptoms caused by *Bacillus cereus* are abdominal pain, vomiting and diarrhea which are very similar to that caused by *Clostridium perfringens* [62,63]. Moreover, it is more difficult to distinguish from other short-term symptoms caused by deterioration (such as those caused by *Staphylococcus aureus*) [64,65]. So, developing an accurate detection method of *Bacillus cereus* in our food is quite significant.

A *B. cereus* electrochemical biosensor based on DNA-based Au nanoparticle modified pencil graphite electrode (PGE) was developed by Soleimanian-Zad et al. [66]. The target was captured by the sensing element comprisinggold nano-particles (GNPs) self-assembled with single-stranded DNA of nheA gene immobilized with thiol linker on the GNPs-modified PGE. The researchers also detected the bacteria in milk and infant formula, which showed that the biosensor was suitable for food safety and quality control applications [13]. Liang et al. [67] published a novel *B. cereus* electrochemical sensor using monoclonal antibodies of *B. cereus* immobilized on double-layer gold nanoparticles to capture the target, and chitosan was used to link the sensing element with GCE. The sensor displayed a fast detection response, long-term stability and high sensitivity to bacterial contamination. A label-free electrochemical biosensor for *Bacillus anthracis* spores was fabricated by Amine et al. [68] using pyrrole to modify the electrode and [Fe(CN)_6_]^3−/4−^ as redox probe.

### 3.4. Staphylococcus aureus

*Staphylococcus aureus* (*S. aureus*) is a typical gram-positive bacterium which could lead to serious purulent infection in human beings, causing pneumonia, pseudomembranous colitis, pericarditis, and even systemic infections such as sepsis [69,70,71]. Food poisoning caused by *Staphylococcus aureus* enterotoxin accounts for between 33% and 45% of all bacterial food poisoning in the United States and Canada, respectively [65,72]. There are also numerous poisoning incidents in China [73,74,75,76].

M. Pingarro’n et al. [77] developed an amperometric biosensor for the quantification of *S. aureus* based on rabbit immunoglobulin (RbIgG) immobilized onto the 3-mercaptopropionic acid (MPA) modified electrode. Using the competitive effect between protein A-bearing *S. aureus* cells and anti-RbIgG labeled with horseradish peroxidase (HRP), the prepared biosensor realized the detection of *S. aureus* in semi-skimmed milk. Subsequently, the research group reported other two electrochemical biosensors for *S. aureus* detection. One is an improvement of previous work which used covalent immobilization for anti-RbIgG at SAM modified gold electrodes by 3, 3′- Dithiodipropionic acid di (N-succinimidyl ester) (DTSP) [78]. Another work took advantage of the MPA-SAM gold electrode modified by RbIgG and tyrosinase [79].

**Figure 3:**
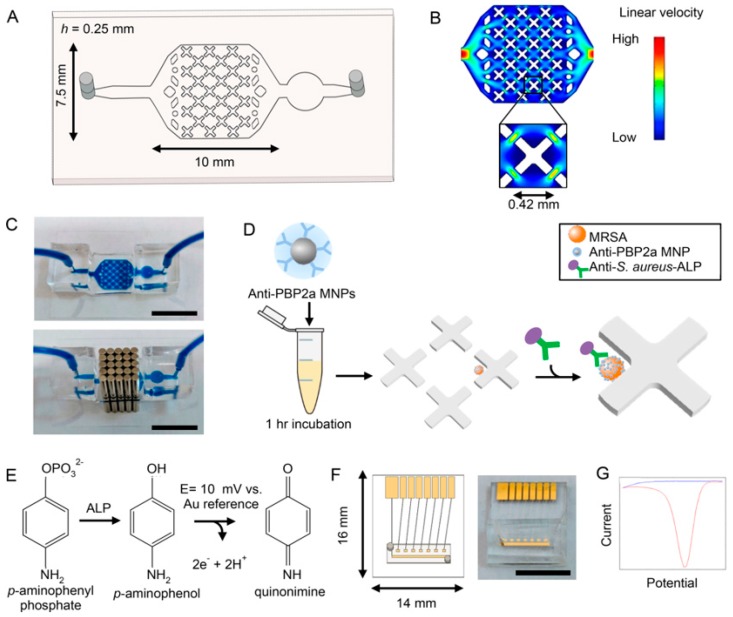
Bacterial capture and electrochemical detection. (**A**) Schematic of bacterial capture device fabricated in PDMS. (**B**) Flow profile of capture device simulated on COMSOL Multiphysics. X-shaped features create areas of reduced flow velocity. (**C**) Photograph of bacterial capture device filled with dye in the absence, and presence of, an array of external magnets (above and below images, respectively). Scale is 10 mm. (**D**) Filtered nasal swab specimen is incubated with anti-PBP2a MNPs for 1 h. The solution is then processed with the device, where magnetically-labeled bacteria are captured in areas of low flow velocity. After wash steps, anti-*S. aureus* antibodies functionalized with ALP are introduced into the device and washed. (**E**) The substrate p-APP is introduced to the device, where it is converted to electrochemically active p-AP by ALP. p-AP is oxidized to quinonimine at a potential of 10 mV against a gold reference electrode. (**F**) Schematic (left) and photograph (right) of electrochemical detector chip. A PDMS channel allows simple transfer of electrochemical readout solution from the capture device. Detection utilizes on-chip working and reference gold electrodes and an external Pt counter electrode. Scale is 10 mm. (**G**) Differential pulse voltammogram displaying signal from p-APP (blue) and p-AP (red). The measured current correlates to number of captured bacteria. Reprinted with permission from [83]. Copyright (2019) American Chemical Society.

Wei et al. [80] reported an electrochemical sensor for *S. aureus* detection using single-stranded DNA as aptamer linked to reduced graphene oxide-gold nanoparticles (rGO-AuNP) nanocomposite by impedance spectroscopy. Mansour et al. [81] also detected *S. aureus* by impedance spectroscopy through monitoring the change of resistance before and after the *S. aureus,* recognized by anti-*S. aureus,* immobilized on gold electrode using ferri-/ferrocyanide as redox probe. The developed biosensor was further used to detect stressed and resuscitated pathogens. Recently, a low-cost screen-printed electrode was applied to build an *S. aureus* biosensor by Connolly et al. [82] using impedance spectroscopy. The targets were incubated in chambers containing the electrodes, and the results analyzed through a novel approach. Impedance spectroscopy provides a label-free method; however, its detection limit is still not low enough compared to other electrochemical biosensors. Methicillin-resistant *S. aureus* collected from patient nasal swabs was captured and detected using a microfluidic device and antibody-functionalized magnetic nanoparticles. As displayed in Figure 3, the identification of *S. aureus* is realized by the use of a strain-specific antibody functionalized with alkaline phosphatase for electrochemical detection [83].

### 3.5. Clostridium Perfringens

*Clostridium perfringens* (*C. perfringens*) is the most common type of *Clostridium* in clinically genital gangrene pathogens. *C. perfringens* can break down sugar in muscle and connective tissue and then release a large amount of gas, which results in severe emphysema of the tissue and affects the supply of blood, ultimately causing a large area of tissue necrosis. The bacterial was named of *C. perfringens* also due to the bacteria can form a capsule in the body [84].

The detection of *clostridium perfringens* by electrochemical method is mainly owing to its DNA. Pu et al. [85] published an electrochemiluminescence sensor for detection of DNA of *C. perfringens* using RCA, like the work of Huang et al. [86]. This research team reported another *Clostridium perfringens* DNA biosensor based on screen-printed electrodes in the same year [64]. They used the stable hairpin form of the initial molecular beacon, which will open after incubating with target DNA, and then the streptavidin aptamer is reactivated. The electrochemical signal of DPV could be detected by “sandwich” reaction. Recently, Wang et al. [87] described an electrochemical biosensor for the detection of DNA of *C. perfringens* based on CeO_2_/chitosan-modified electrodes by monitoring the changes of impedance.

### 3.6. Simultaneous Detection of Multiple Foodborne Pathogens

There seems to be a trend of developing the electrochemical biosensor for the simultaneous and multiple detection of biologically pathogens [88]. A multi-junction sensor was constructed for potential multiplexed detection of *E. coli* and *S. aureus* based on a 2 × 2 junction array formed with gold tungsten wires on single walled carbon nanotube and polyethylenimine. The detection time is rapid and the LODs for *E. coli* and *S. aureus* were 10 μL and 100 μL, respectively [89]. Li et al. [57] developed a sandwich-type electrochemical biosensor based on Au/GCP for simultaneous ultrasensitive detection of *E. coli* O157:H7 and *Vibrio cholerae* O1. The detection antibodies specific for *E. coli* O157:H7 and *Vibrio cholerae* O1 were labeled by CdS and PbS nanoparticles via C60@AuNPs as nanocarriers and HCR amplification, respectively. The antibodies used for capture pathogens were linked to streptavidin-coated magnetic beads (MB@SA). The prepared biosensor displayed excellent performance and this method could be expanded readily for detecting other pathogenic bacteria and would be of great value for future applications in food safety. Furthermore, Ai et al. [90] built an efficient electrochemical disinfection for *E. coli* and *S. aureus* in drinking water based on ferrocene–PAMAM–multi-walled carbon nanotubes–chitosan nanocomposite modified pyrolytic graphite electrode. When applying a potential of 0.4 V for 10 min, almost all pathogens were killed, demonstrating that they provided a valid electrochemical method for the disinfection of pathogens.

## 4. Conclusions and Perspective

Although some traditional methods for detection of foodborne pathogens are sensitive, most of them are also time-consuming (a few days to a week), which limit their practical application. Therefore, developing new methods to detect foodborne pathogens is necessary. Electrochemical biosensing technology has been maturely applied to the rapid determination of pathogens through exploration and development.

Electrochemical biosensors based on nucleic acid or aptamer displayed high sensitivity and low detection limit, however the stability and accuracy should be improved. The electrochemical biosensor based on the combination between antigen and antibody is a big family of biosensors used for the detection of pathogens. These biosensors have high accuracy, but the detection limit is not low enough, especially the biosensors based on the sandwiched principle. The tendency for electrochemical biosensors of pathogens is that multiple pathogens were detected simultaneously. In summary, there is room for further improvement for the detection methods for food pathogens. A rapid, sensitive and low-cost detection method for foodborne pathogens has a huge market prospect. Given the demand and preponderance of electrochemical sensing, there is still a great chance for further developments in the detection of food pathogens in the near future.

## Figures and Tables

**Table 1:** Biosensors for the detection of foodborne pathogens.

Analyst	Detection Type	Materials	Performance	Reference
*E. coli*	Amperometric	screen-printed electrode	Rapid determination of four *E. coli* subspecies Assay time: approximately 2 min	[46]
*E. coli*	Amperometric	DNA nanopyramids	Linear range: 1–10^2^ CFU/mL LOD: 1.20 CFU/mL	[47]
*E. coli*	Amperometric	G-quadruplex/hemin/Gold electrode	Linear range: 9.4–9.4 × 10^5^ CFU/mL LOD: 8 CFU/mL	[86]
*E. coli*	Impedimetric	rGO-CysCu/Gold electrode	Linear range: 100–10^8^ CFU/mL LOD: 3.8 CFU/mL Assay time: > 1 h	[48]
*E. coli*	Impedimetric	BSA-conjugated 3D Ag nanoflowers	Linear range: 3.0 × 10^2^–3.0 × 10^8^ CFU/mL LOD: 100 CFU/mL	[49]
*E. coli*	Amperometric	T7_lacZ_ phages/PAGE	10^5^ CFU/mL in 3 h and 10^2^ CFU/mL after 7 h	[50]
*E. coli*	Amperometric	CdS@ZIF-8 particles	Linear range: 10–10^8^ CFU/mL Assay time: < 3 h LOD: 3 CFU/mL (S/N=3)	[51]
*Vibrio cholerae*	Amperometric	ALP/screen-printed electrodes	LOD: 10^5^ cells/mL Assay time: < 55 min	[55]
*Vibrio cholerae*	Amperometric	screen-printed electrodes	8 CFU/mL in sea water, 80 CFU/mL sewer water and tap water Assay time: 55 min	[56]
*Vibrio cholera*	Amperometric	Biotinylated-PAb/ SPE	LOD: 4 × 10^2^ cells/mL Assay time: < 1 h	[57]
*Vibrio cholerae*	Impedimetric	CeO_2_ nanowire-modified microelectrode	Linear range: 1.0 × 10^2^–1.0 × 10^4^ CFU/mL	[58]
*B. cereus*	Impedimetric	GNPs-sDNA-(nheA)/PGE	Sensitivity: 10^0^ CFU/mL LOD: 9.4 × 10^−12^ mol/L	[66]
*B. cereus*	Amperometric	GNPs-Chit-GCE	Linear range: 5.0 × 10^1^ to 5.0 × 10^4^ CFU/mL LOD: 10.0 CFU/mL (S/N = 3)	[67]
*B. cereus*	Potentiometric	CPE/SIP	Linear range: 10^2^–10^5^ CFU/mL	[68]
*S. aureus*	Amperometric	HRP-MPA/gold electrode	LOD: 1.6 × 10^5^ cells/mL	[77]
*S. aureus*	Amperometric	HRP-DTSP-/Screen-printed electrodes	Linear range: 1.3 × 10^3^–7.6 × 10^4^ cells/mL LOD: 3.7 × 10^2^ cells/mL Assay time: approximately 30 min	[78]
*S. aureus*	Amperometric	AP-MPA/gold electrode	Linear range: 4.4 × 10^5^–1.8 × 10^7^ cells/mL LOD: 1.7 × 10^5^ cells/mL Assay time: approximately 25 min	[79]
*S. aureus*	Impedimetric	Aptamer/rGO-AuNP/GCE	Linear range:10–10^6^ CFU/mL LOD: 10 CFU/mL (S/N=3) Assay time: < 1 h	[80]
*S. aureus*	Impedimetric	MPA/gold electrode	Linear range: 10^1^–10^7^ CFU/mL LOD: 10 CFU/mL	[81]
*S. aureus*	Impedimetric	screen printed electrode	Linear range: 3.6 × 10^7^–9.3 × 10^7^ CFU/mL Assay time: approximately 30 min	[82]
DNA of *C. perfringens*	Electrochemiluminescence	gold electrode (rolling circle amplification)	LOD: 10^−15^ M Assay time: approximately 1 h	[85]
DNA of *C. perfringens*	Amperometric	SA/ADH/Fe_3_O_4_ nanocomposites	Linear range: 10^−12^–10^−6^ M Assay time: same as PCR	[64]
*C. perfringens*	Impedimetric	CeO2/chitosan/GCE	Linear range: 1.0 × 10^−14^–1.0 × 10^−7^ mol/L LOD: 7.06 × 10^−15^ mol/L	[87]

CPE: carbon paste electrode; SIP: spore-imprinted polymer; HRP: horseradish peroxidase; MPA: 3-mercaptopropionic acid; ADH: alcohol dehydrogenase.
